# Trends in the incidence of influenza and influenza-like illness in Cambodia, 2006–2023: insights from national sentinel surveillance

**DOI:** 10.5365/wpsar.2026.17.2.1132

**Published:** 2026-06-30

**Authors:** Sokly Mom, Khanuengnij Yueayai, Seng Heng, Hay Puthik Long, Sopheavy Seng, Nathaprang Nittayasoon, Sengdoeurn Yi, Sovann Ly

**Affiliations:** a International Field Epidemiology Training Program, Ministry of Public Health, Nonthaburi, Thailand.; b Department of Communicable Disease Control, Ministry of Health, Phnom Penh, Cambodia.; c Cambodia Field Epidemiology Training Program, Department of Communicable Disease Control, Ministry of Health, Phnom Penh, Cambodia.; d Division of Epidemiology, Department of Disease Control, Ministry of Public Health, Nonthaburi, Thailand.

## Abstract

Understanding the patterns and occurrence of influenza is crucial for effective public health responses. National sentinel surveillance systems provide invaluable data that can be used to track the prevalence, seasonality and evolution of influenza viruses, and to inform timely interventions and early warning systems for disease control. This study aimed to describe the trends in influenza-like illness (ILI) and influenza incidence based on data obtained from ILI surveillance conducted among patients presenting to health facilities with symptoms indicative of influenza in Cambodia during 2006–2023. A total of 27 098 samples were tested, of which 15.5% were positive for influenza. Of these, 63.6% were influenza A, 36.2% were influenza B, and 0.2% were both influenza A and B. The annual incidence of influenza among the outpatient population varied, averaging 8.2 cases (range: 0.05–23.8) per 1000 population. Incidence rates were highest in 2007, 2008 and early 2023. The overall positivity rate was 16%. Not all ILI surveillance sites were consistently active during the study period, primarily due to funding constraints. Given the constraints of sentinel site coverage and data collection inconsistency, the study emphasizes the vital need for ongoing and improved ILI surveillance to accurately assess influenza incidence. To gain a more detailed picture of influenza dynamics in Cambodia, surveillance systems should be strengthened and data collection should be expanded to include asymptomatic patients and other important patient characteristics. These results are needed to guide regional and national influenza preparedness, prevention and control initiatives.

Influenza A and B have driven worldwide epidemics both historically and recently. (*1*) According to World Health Organization (WHO) estimates, 3–5 million severe influenza cases and 290 000–650 000 deaths occur annually worldwide. (*1*) Studies conducted between 2006 and 2016 suggest influenza has a substantial public health burden in Cambodia, with a high prevalence of both influenza A and B and influenza positivity in the range of 8–16%. (*2*-*4*) Furthermore, analysis of national data for 2015–2016 has revealed consistently high influenza hospitalization rates across all age groups, averaging 56 cases per 100 000 population. (*4*)

The Department of Communicable Disease Control of Cambodia’s Ministry of Health (CCDC) plays a lead role in the response and control of infectious diseases, including influenza. While influenza is a year-round endemic disease in Cambodia, infection rates peak during the June–November rainy season. Surveillance mechanisms for influenza-like illness (ILI) were established in 2006 in collaboration with WHO, the United States Centers for Disease Control and Prevention (US CDC) and other health partners. Since 2014, Cambodia has been a recipient of Partnership Contributions through the WHO Pandemic Influenza Preparedness Framework, which have enabled the country to strengthen capacities in influenza surveillance, laboratory capacity and pandemic preparedness at the national and provincial levels. (*5*) Cambodia currently contributes data to WHO’s Global Influenza Surveillance and Response System. (*6*) Cambodia’s ILI surveillance monitoring network has since proved invaluable for responding to national influenza outbreaks and for tracking the emergence of individual subtypes and strains, in relation to both seasonal influenza and avian influenza. (*7*, *8*)

This study aimed to describe trends in the incidence of ILI and influenza in Cambodia over an extended period, 2006–2023, based on data obtained from Cambodia’s sentinel ILI surveillance network. Understanding the trends in influenza and ILI incidence through sentinel surveillance data remains crucial for enhancing Cambodia’s capacity to respond effectively to seasonal influenza outbreaks and has direct implications for national public health planning. This data analysis also contributes to the global effort to improve influenza surveillance and preparedness.

## Methods

### Study design and setting

**Fig. 1:**
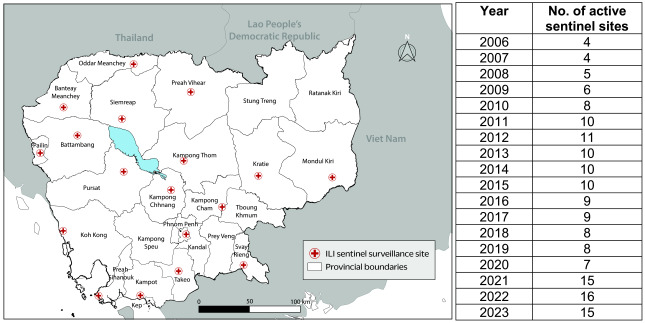
Location and number of ILI sentinel surveillance sites across Cambodia, June 2006–July 2023

This cross-sectional study analysed retrospective data collected by the ILI surveillance system in Cambodia between June 2006 and July 2023. During the study period, 18 sentinel sites for ILI surveillance were in operation across the country (**Fig. 1**). The sites comprised a mix of health centres, referral hospitals and national hospitals; two sites were located at paediatric hospitals that treat only patients under the age of 16 years. Routine operation of ILI surveillance was not consistent across all sites throughout the study period due to resource constraints and protocol modifications (**Fig. 1**).

[**insert** Figure 1]

### Data sources

CCDC manages the ILI surveillance system in collaboration with multiple agencies such as the National Institute of Public Health (NIPH) and the Institut Pasteur du Cambodge (IPC), which provide laboratory testing services. Data obtained by CCDC from its network of ILI surveillance sites for the period from June 2006 to July 2023 were used for analysis. Weekly aggregate ILI data are reported from sentinel surveillance sites, including the number of ILI cases and total outpatients. In cases where a sample is collected for testing, individual-level data with laboratory results are reported to CCDC.

The extracted ILI surveillance data included the total number of outpatients, the number of ILI patients, the number of specimens collected from ILI patients for laboratory testing and the number of influenza-positive individuals. Extracted data also included demographic characteristics (age, sex), self-reported signs and symptoms (including fever, cough, runny nose, sore throat, headache, vomiting/nausea, body aches and diarrhoea), the date of symptom onset and the date of sample collection.

### Case definition and laboratory testing

The ILI case definition is a person presenting with sudden onset of fever (≥ 38.0 °C measured by axillary temperature) with cough and/or sore throat in the absence of another diagnosis. Approximately 5–10 nasopharyngeal swabs from patients presenting with ILI per site per week were collected for laboratory testing. The ILI case definition and the frequency of sample collection varied over the course of the study due to minor protocol changes. Influenza diagnosis was confirmed by a positive test result for influenza A or B viruses using multiplex real-time polymerase chain reaction (RT–PCR). Then, specimens confirmed positive for influenza A and/or B were extracted, and RNA was isolated to determine the virus subtype using a singleplex RT–PCR method following the US CDC protocol for detecting and characterizing seasonal influenza viruses. Influenza A and B testing was performed by NIPH, and IPC reconfirmed all positive specimens and 10% of negative specimens.

### Descriptive data analysis

The data were extracted from the ILI surveillance database into Microsoft Excel. Data on demographic characteristics were categorized for analysis. Age was categorized as < 1 year, 1–4 years, 5–14 years, 15–24 years, 25–49 years and ≥ 50 years. Sex was categorized as male and female. Clinical symptoms were categorized as “yes,” “no” and “don’t know” based on patient reports; “don’t know” was treated as missing data. Time to diagnosis was categorized into two groups, early diagnosis (≤ 2 days) and diagnostic delay (> 2 days), calculated as the time from the patient-reported date of symptom onset to the date of sample collection.

### Statistical analysis

The influenza positivity rate was calculated by dividing the total number of specimens that tested positive for influenza by the total number of specimens tested for influenza. ILI incidence (per 1000 population) was determined by dividing the number of outpatients presenting with ILI symptoms by the total number of outpatients. Influenza incidence (per 1000 population) was calculated using the methodology adopted by Fowlkes et al. (*9*) Influenza incidence was calculated by multiplying the positivity rate (number of cases positive for influenza by PCR test divided by the total number of tested cases) by the total number of ILI cases who visited a health facility as an outpatient, and then dividing this by the total number of outpatient visits as the population denominator. Sentinel ILI surveillance sites were categorized by province. Data analyses were carried out using Stata version 14 (StataCorp 77845, College Station, TX, USA).

## Results

### Characteristics of ILI cases

**Fig. 2:**
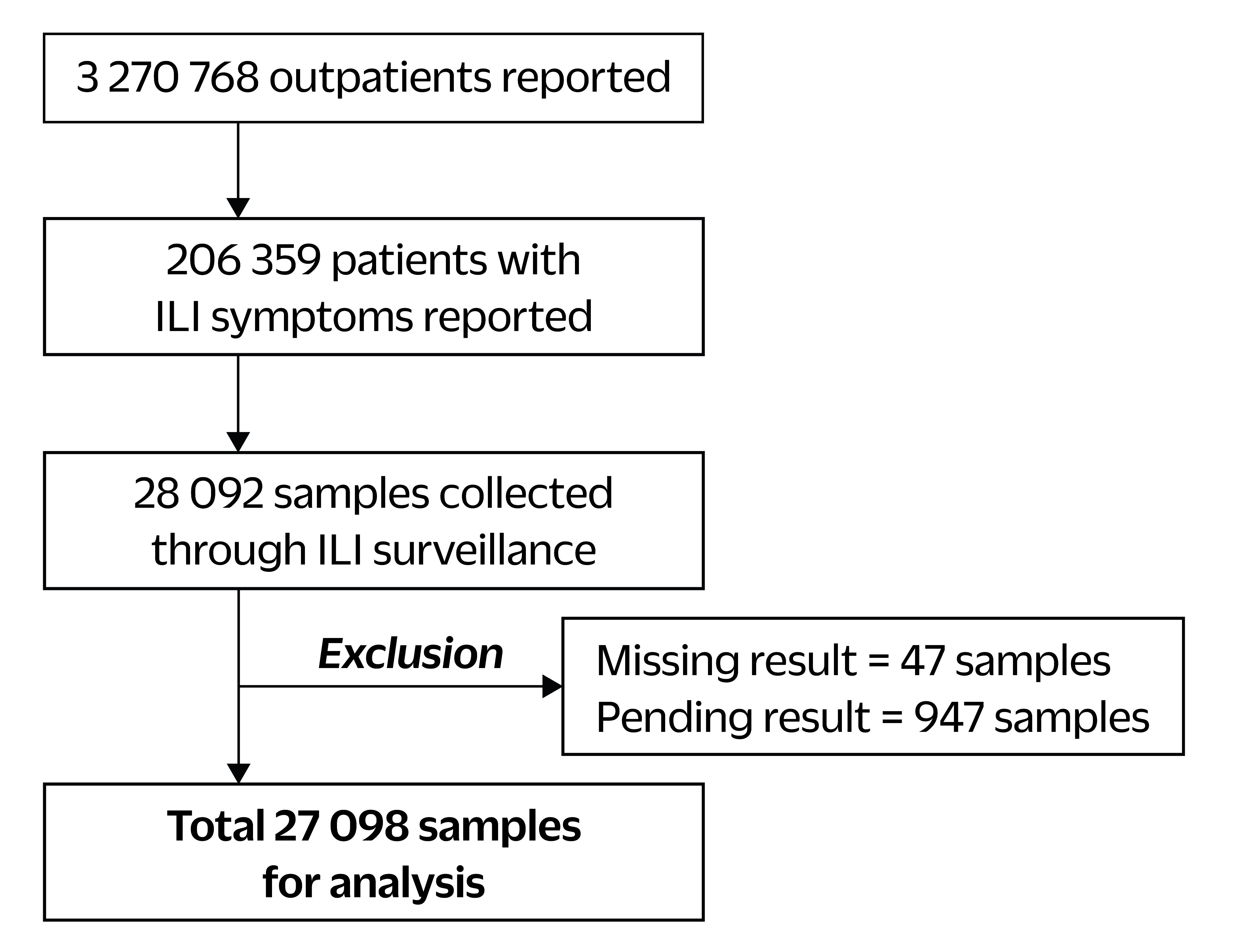
Inclusion and exclusion criteria for data analysis of the samples collected from ILI surveillance, Cambodia, June 2006–July 2023

Between June 2006 and July 2023, a cumulative total of 3 270 768 people visited ILI sentinel sites as outpatients, of whom 206 359 (6.3%) presented with ILI symptoms. A total of 28 092 samples were collected for PCR testing, of which 27 098 (96.5%) were tested, excluding 994 with missing or pending results (**Fig. 2**). Of the tested specimens, 4202 (15.5%) were positive for various influenza subtypes (**Table 1**).

[**insert** Figure 2]

**Table 1:** Total specimens, influenza-positive specimens and influenza subtypes by year, Cambodia, June 2006–July 2023

Year	Total specimens	No. (%) of influenza-positive specimens	No. (%) of influenza viruses by subtype or lineage								
			A (not subtyped)	A(H1N1)pdm	A(H3N2)	A(H5N1)	A(H3N2) and A(H1N1)pdm	A and B	B (lineage not determined)	B/Victoria	B/Yamagata
**Total**	**27 098**	**4202 (15.5)**	**6 (0.1)**	**1019 (24.3)**	**1644 (39.1)**	**1 (0.02)**	**1 (0.02)**	**8 (0.2)**	**967 (23.0)**	**390 (9.3)**	**166 (4.0)**
**Jun–Dec 2006**	**474**	**21 (4.4)**	**–**	**–**	**21 (100)**	**–**	**–**	**–**	**–**	**–**	**–**
**2007**	**1218**	**91 (7.5)**	**4 (4.4)**	**11 (12.1)**	**25 (27.5)**	**–**	**–**	**2 (2.2)**	**49 (53.8)**	**–**	**–**
**2008**	**1360**	**214 (15.7)**	**–**	**26 (12.1)**	**115 (53.7)**	**–**	**–**	**–**	**73 (34.1)**	**–**	**–**
**2009**	**2319**	**400 (17.2)**	**–**	**93 (23.3)**	**215 (53.8)**	**–**	**–**	**–**	**92 (23.0)**	**–**	**–**
**2010**	**2577**	**387 (15.0)**	**–**	**134 (34.6)**	**153 (39.5)**	**–**	**–**	**–**	**100 (25.8)**	**–**	**–**
**2011**	**2808**	**571 (20.3)**	**–**	**139 (24.3)**	**53 (9.3)**	**–**	**–**	**4 (0.7)**	**375 (65.7)**	**–**	**–**
**2012**	**2805**	**344 (12.3)**	**–**	**20 (5.8)**	**238 (69.2)**	**–**	**–**	**–**	**86 (25.0)**	**–**	**–**
**2013**	**1558**	**259 (16.6)**	**–**	**66 (25.5)**	**18 (6.9)**	**1 (0.4)**	**–**	**2 (0.8)**	**172 (66.4)**	**–**	**–**
**2014**	**1554**	**189 (12.2)**	**–**	**18 (9.5)**	**155 (82.0)**	**–**	**1 (0.5)**	**–**	**15 (7.9)**	**–**	**–**
**2015**	**1549**	**262 (16.9)**	**–**	**18 (6.9)**	**195 (74.4)**	**–**	**–**	**–**	**1 (0.4)**	**1 (0.4)**	**47 (17.9)**
**2016**	**1524**	**397 (26.0)**	**–**	**137 (34.5)**	**50 (12.6)**	**–**	**–**	**–**	**4 (1.0)**	**193 (48.6)**	**13 (3.3)**
**2017**	**1069**	**130 (12.2)**	**–**	**40 (30.8)**	**83 (63.8)**	**–**	**–**	**–**	**–**	**4 (3.1)**	**3 (2.3)**
**2018**	**1262**	**214 (17.0)**	**–**	**106 (49.5)**	**3 (1.4)**	**–**	**–**	**–**	**–**	**16 (7.5)**	**89 (41.6)**
**2019**	**1201**	**276 (23.0)**	**–**	**130 (47.1)**	**52 (18.8)**	**–**	**–**	**–**	**–**	**80 (29.0)**	**14 (5.1)**
**2020**	**1286**	**199 (15.5)**	**–**	**17 (8.5)**	**182 (91.5)**	**–**	**–**	**–**	**–**	**–**	**–**
**2021**	**1097**	**2 (0.2)**	**2 (100)**	**–**	**–**	**–**	**–**	**–**	**–**	**–**	**–**
**2022**	**1025**	**170 (16.6)**	**–**	**2 (1.2)**	**85 (50.0)**	**–**	**–**	**–**	**–**	**83 (48.8)**	**–**
**Jan–Jul 2023**	**412**	**76 (18.4)**	**–**	**62 (81.6)**	**1 (1.3)**	**–**	**–**	**–**	**–**	**13 (17.1)**	**–**

Among the positive cases, 2671 (63.6%) were attributed to influenza A, 1523 (36.2%) to influenza B and 8 (0.2%) to both influenza A and B (**Table 1**). The median age of the positive cases was 2.3 years (interquartile range: 1–8 years).

Positivity varied by age group; for children aged < 1 year, the positivity rate was 10.3% (687/6672), rising to 15.6% (1626/10 428) in those aged 1–4 years, and peaking at 25.7% (1401/5446) in those aged 5–14 years. The rate dropped back to 16.5% (227/1373) in those aged 15–24 years, and to < 10% in the two oldest age groups (8.8% [202/2308] in those aged 25–49 years, 6.8% [59/871] in those aged ≥ 50 years). Positivity was similar between the sexes; 14.8% (1928/13 068) of females and 16.2% (2274/14 030) of males tested had a positive result for influenza (**Table 2**).

**Table 2:** Influenza positivity and subtypes by age and sex, Cambodia, June 2006–July 2023

Characteristic	Total outpatients	No. (%) of patients with ILI	Total specimens	No. (%) of influenza-positive specimens	No. (%) of influenza viruses by subtype or lineage								
					A (not subtyped)	A(H1N1)pdm	A(H3N2)	A(H5N1)	A(H3N2) and A(H1N1)pdm	A and B	B (lineage not determined)	B/Victoria	B/Yamagata
**Total**	**3 270 768**	**206 359 (6.3)**	**27 098**	**4202 (15.5)**	**6 (0.1)**	**1019 (24.3)**	**1644 (39.1)**	**1 (0.02)**	**1 (0.02)**	**8 (0.2)**	**967 (23.0)**	**390 (9.3)**	**166 (4.0)**
**Age group, years**													
** < 1**	**258 494**	**11 538 (4.5)**	**6672**	**687 (10.3)**	**1 (0.1)**	**188 (27.4)**	**305 (44.4)**	**1 (0.1)**	**–**	**–**	**145 (21.1)**	**30 (4.4)**	**17 (2.5)**
**1–4**	**1 382 839**	**115 136 (8.3)**	**10 428**	**1626 (15.6)**	**2 (0.3)**	**368 (22.6)**	**709 (43.6)**	**–**	**1 (0.1)**	**1 (0.1)**	**322 (19.8)**	**163 (10.0)**	**60 (3.7)**
**5–14**	**933 395**	**64 982 (7.0)**	**5446**	**1401 (25.7)**	**3 (0.1)**	**335 (23.9)**	**425 (30.3)**	**–**	**–**	**5 (0.4)**	**402 (28.7)**	**160 (11.4)**	**71 (5.1)**
**15–24**	**155 143**	**2341 (1.5)**	**1373**	**227 (16.5)**	**–**	**64 (28.2)**	**90 (39.6)**	**–**	**–**	**2 (0.9)**	**43 (18.9)**	**21 (9.3)**	**7 (3.1)**
**25–49**	**352 769**	**9394 (2.7)**	**2308**	**202 (8.8)**	**–**	**46 (22.8)**	**88 (43.6)**	**–**	**–**	**–**	**44 (21.8)**	**14 (6.9)**	**10 (5.0)**
** ≥ 50**	**188 128**	**2968 (1.6)**	**871**	**59 (6.8)**	**–**	**18 (30.5)**	**27 (45.8)**	**–**	**–**	**–**	**11 (18.6)**	**2 (3.4)**	**1 (1.7)**
**Sex**													
**Female**	**–**	**–**	**13 068**	**1928 (14.7)**	**2 (0.1)**	**470 (24.4)**	**757 (39.3)**	**–**	**–**	**6 (0.3)**	**452 (23.4)**	**165 (8.6)**	**76 (3.9)**
**Male**	**–**	**–**	**14 030**	**2274 (16.3)**	**4 (0.2)**	**549 (24.1)**	**887 (39.0)**	**1 (0.04)**	**1 (0.04)**	**2 (0.1)**	**515 (22.6)**	**225 (9.9)**	**90 (4.0)**

ILI: influenza-like illness.

The 18 sentinel sites were active for a mean of 7.8 years (range: 1–18 years) during the 18-year study period. Those that were active for longer tended to report more ILI cases, which ranged from 22 at a site that was active for 2 years to 126 002 at a site that was active for 16 years. The percentage of influenza-positive patients at each site ranged from 0% to 13.5%. The two paediatric hospitals cumulatively saw more patients with ILI symptoms (78.3%) and influenza-positive patients (51.1%) than all other sites combined (**Table 3**).

**Table 3:** ILI and influenza-positive patients by sentinel site active during the study period, Cambodia,  June 2006–July 2023

ILI surveillance site	No. of patients with ILI symptoms	No. (%) of influenza-positive patients	No. of years (year range) as an active ILI sentinel site
**National Paediatric Hospital^a^**	**126 002**	**929 (0.7)**	**16 (2008–2023)**
**Angkor Hospital for Children^a^**	**35 590**	**1218 (3.4)**	**18 (2006–2023)**
**Battambang Hospital**	**13 908**	**417 (3.0)**	**15 (2006–2017, 2021–2023)**
**Kampong Cham Hospital**	**13 664**	**451 (3.3)**	**18 (2006–2023)**
**Takeo Hospital**	**5715**	**131 (2.3)**	**7 (2006–2012)**
**Mondulkiri Hospital**	**2664**	**356 (13.4)**	**14 (2010–2023)**
**Kampot Hospital**	**2528**	**275 (10.9)**	**14 (2010–2023)**
**Svay Rieng Hospital**	**2363**	**263 (11.1)**	**15 (2021–2023)**
**Anlong Veng Hospital**	**1541**	**32 (2.1)**	**1 (2011)**
**Paillin Hospital**	**743**	**100 (13.5)**	**8 (2011–2017, 2022)**
**Koh Kong Hospital**	**643**	**1 (0.2)**	**3 (2020–2022)**
**Kratie Hospital**	**302**	**22 (7.3)**	**2 (2022–2023)**
**Pursat Hospital**	**234**	**1 (0.2)**	**2 (2020–2021)**
**Preash Vihear Hospital**	**178**	**0 (0)**	**2 (2021–2022)**
**Tbong Kmum Hospital**	**95**	**0 (0)**	**1 (2021)**
**Banteay Meanchey Hospital**	**86**	**0 (0)**	**1 (2011, 2013)**
**Sihanukvill Hospital**	**81**	**4 (4.9)**	**2 (2020–2021)**
**Kampong Thom Hospital**	**22**	**2 (9.1)**	**2 (2020–2021)**
**Total**	**206 359**	**4202 (15.5)**	**18 (2006–2023)**

ILI: influenza-like illness.

^a^ Hospitals that treat children and adolescents.

The most frequently reported symptoms among influenza-positive cases were fever (99.7%), cough (98.7%) and runny nose (86.3%). Of positive cases with a recorded date of diagnosis, 78.1% (2506/3208) were diagnosed in ≤ 2 days and 702 (21.9%) were diagnosed in > 2 days (**Table 4**).

**Table 4:** Clinical symptoms and time to diagnosis in ILI patients who were tested for influenza and influenza-positive patients, Cambodia, June 2006–July 2023

Variable	Tested for influenza, *n*(%)	Influenza-positive, *n*(%)
**Clinical symptom**	***n* = *27 098***	***n* = *4 202***
**Fever^a^**	**26 922 (99.4)**	**4190 (99.7)**
**Cough**	**26 384 (97.4)**	**4146 (98.7)**
**Runny nose**	**21 877 (80.7)**	**3628 (86.3)**
**Sore throat**	**10 887 (40.2)**	**1994 (47.5)**
**Headache**	**6146 (22.7)**	**1417 (33.7)**
**Vomiting/nausea**	**5287 (19.5)**	**931 (22.2)**
**Body aches**	**2989 (11.0)**	**690 (16.4)**
**Diarrhoea**	**2921 (10.8)**	**349 (8.3)**
**Other symptoms**	**497 (1.8)**	**78 (1.9)**
**Time to diagnosis^b^**	***n^c^ = 20 241***	***n^c^ = 3 208***
**Early diagnosis (≤ 2 days)**	**15 390 (76.0)**	**2506 (78.1)**
**Diagnostic delay (> 2 days)**	**4851 (24.0)**	**702 (21.9)**

^a^ A total of 122 (116/27 098 and 6/4202) specimens were missing data for fever.

^b^ The median (interquartile range) time to diagnosis was 2 (1–2) days.

^c^ Cases with a missing diagnosis date were excluded from the total.

### Estimated incidence and positivity rate of ILI and influenza

**Fig. 3:**
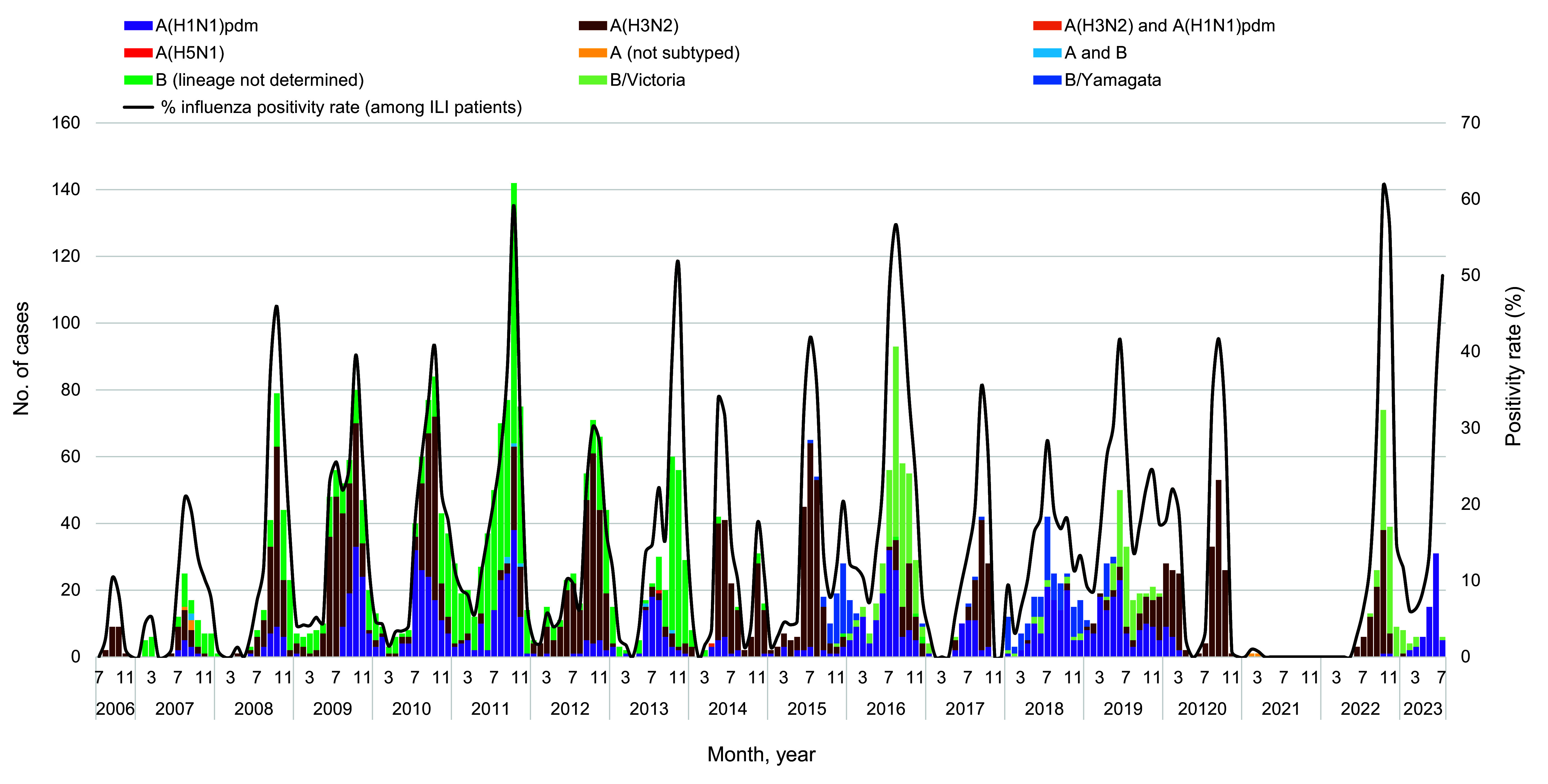
Trends in influenza cases by subtype and lineage detected by ILI surveillance sites, Cambodia, June 2006–July 2023

The estimated incidence of ILI among the outpatient population for the entire study period was 52.6 cases per 1000 population (**Table 5**). The annual incidence ranged from 23.3 to 191.2 cases per 1000 population. The highest annual incidence rates occurred in 2007 and 2008, which were more than double all other years. The estimated incidence of influenza for the entire study period was 8.2 per 1000 population. The annual incidence ranged from 0.05 to 23.8 per 1000 population, and rates were highest in 2007, 2008 and early 2023. Influenza trends were consistently high between May and December in most years. Monthly trends in influenza cases, categorized by subtype and showing clear seasonal peaks, are illustrated in **Fig. 3**.

[**insert** Figure 3]

**Table 5:** Trends in estimated ILI and influenza incidence among outpatients, Cambodia, June 2006–July 2023

Year	No. of ILI sites	No. of outpatients	No. (%) of outpatients with ILI symptoms	No. of specimens tested	No. (%) of influenza-positive specimens	Estimated incidence per 1 000 outpatients^a^	
						ILI symptoms	Influenza
**Total**	**18**	**3 921 609**	**206 359 (5.3)**	**27 098**	**4202 (15.5)**	**52.6**	**8.2**
**Jun–Dec 2006^b^**	**4**	**217 867**	**13 311 (6.1)**	**474**	**21 (4.4)**	**61.1**	**2.7**
**2007^b^**	**4**	**217 867**	**41 663 (19.1)**	**1218**	**91 (7.5)**	**191.2**	**14.3**
**2008^b^**	**5**	**217 867**	**32 966 (15.1)**	**1360**	**214 (15.7)**	**151.3**	**23.8**
**2009**	**6**	**220 592**	**11 596 (5.3)**	**2319**	**400 (17.2)**	**52.6**	**9.1**
**2010**	**8**	**230 593**	**9817 (4.3)**	**2577**	**387 (15.0)**	**42.6**	**6.4**
**2011**	**10**	**231 824**	**10 511 (4.5)**	**2808**	**571 (20.3)**	**45.3**	**9.2**
**2012**	**11**	**256 927**	**9731 (3.8)**	**2805**	**344 (12.3)**	**37.9**	**4.6**
**2013**	**10**	**209 589**	**8723 (4.2)**	**1558**	**259 (16.6)**	**41.6**	**6.9**
**2014**	**10**	**219 847**	**8236 (3.7)**	**1554**	**189 (12.2)**	**37.5**	**4.6**
**2015**	**10**	**228 821**	**9255 (4.0)**	**1549**	**262 (16.9)**	**40.4**	**6.8**
**2016**	**9**	**270 523**	**10 112 (3.7)**	**1524**	**397 (26.0)**	**37.4**	**9.7**
**2017**	**9**	**221 831**	**6976 (3.1)**	**1069**	**130 (12.2)**	**31.4**	**3.8**
**2018**	**8**	**266 148**	**6190 (2.3)**	**1262**	**214 (17.0)**	**23.3**	**3.9**
**2019**	**8**	**278 769**	**7938 (2.8)**	**1201**	**276 (23.0)**	**28.5**	**6.5**
**2020**	**7**	**184 829**	**5031 (2.7)**	**1286**	**199 (15.5)**	**27.2**	**4.2**
**2021**	**15**	**157 914**	**4091 (2.6)**	**1097**	**2 (0.2)**	**25.9**	**0.05**
**2022**	**16**	**197 074**	**6006 (3.0)**	**1025**	**170 (16.6)**	**30.5**	**5.1**
**Jan–Jul 2023**	**15**	**92 727**	**4206 (4.5)**	**412**	**76 (18.4)**	**45.4**	**10.3**

ILI: influenza-like illness.

^a^ Estimated influenza incidence per 1000 outpatients = (number of influenza-positive specimens/number of specimens tested) x (number of outpatients with ILI symptoms/number of outpatients).

^b^ Data on outpatient visits were missing for 2006–2008 and were replaced with the average annual number of outpatient visits per site.

## Discussion

This study explored trends in ILI and influenza incidence in Cambodia over an 18-year period from June 2006 to July 2023, based on data collected by the national sentinel surveillance network. Our analysis showed that influenza positivity averaged 15.5% over the study period, a proportion that falls within the range reported in previous studies (8–36%). (*2*-*4*) Influenza positivity was found to be spread across all age groups, with proportions ranging from 6.8% to 25.7% (**Table 2**), in line with these previous studies. However, influenza was more prevalent in individuals aged < 15 years, which aligns with a previous study conducted in Cambodia that examined influenza associated with hospitalized patients from severe acute respiratory infection surveillance. (*4*)

Positivity was similar in females (14.7%) and males (16.3%) and ranged from 6.8% among those aged > 50 years to 25.7% among those aged 5–14 years. Among influenza-positive cases, 63.6% were attributed to various subtypes of influenza A, while 36.2% were subtypes of influenza B and the remaining 0.2% were both A and B, consistent with data from the Asia–Pacific. (*10*)

During the study period, the estimated incidence of ILI was highest in 2007 and 2008 (191.2 and 151.3 per 1000 population, respectively), but since then has ranged between 23.3 and 52.6 per 1000 population. Importantly, the estimated incidence of influenza was consistently low, averaging < 10 per 1000 population across all years. The high incidence observed in 2007 and 2008, followed by a decline in subsequent years, aligns with the cyclical nature of influenza epidemics. The increase in the incidence of ILI and influenza might be attributed to the change in the ILI case definition. (*2*) Other studies conducted in China between 2006 and 2015 revealed similar trends in influenza incidence, except for the influenza A(H1N1) pandemic in 2009, which had a higher incidence. (*11*, *12*)

There was a notable drop in the number of ILI and influenza cases detected in 2021 and early 2022. The demands on laboratory resources (human resource capacity for testing SARS-CoV-2 specimens) meant that influenza virus testing was stalled during the height of the COVID-19 pandemic but resumed in October 2022. This drop mirrors trends seen in many countries and is attributed to the impact of the COVID-19 pandemic, which led to lockdowns globally, including in Cambodia. (*13*)

### Limitations

The study had several limitations. First, the true incidence of influenza was only recorded for individuals with symptoms, and asymptomatic cases were not captured. As a result, the incidence in the study might be underestimated, though we attempted to adjust our analysis by using outpatients as our population denominator in place of the general population. Second, not all sentinel sites were consistently active during the study period, which could have influenced our findings. Some hospitals were not used as ILI surveillance sites due to their small sample size and the limited duration of surveillance and funding constraints. Third, we were unable to obtain data on several potentially relevant variables, such as education level, occupation, and time from onset to discharge from the health facility, preventing us from fully measuring the factors contributing to the outcomes in this study. The limited data completeness at specific sites, coupled with uncertainty regarding whether the data were collected at the health centre level or hospital level, restricted the scope of our analysis in comprehending ILI and influenza trends across all health facilities.

### Conclusion

This study highlights the long-term trends and incidence of ILI and influenza in Cambodia over an 18-year period. The findings reveal that influenza affects all age groups, with the highest positivity rates observed among children aged 5–14 years and a predominance of influenza A subtypes. Despite a consistently low overall incidence of influenza (annual mean of < 10 per 1000 population), significant annual and seasonal variations were observed, with peaks aligning with the rainy season. Not all ILI surveillance sites were consistently active, primarily due to funding constraints. The study underscores the critical need for sustained and enhanced ILI surveillance to capture the true incidence of influenza, particularly given the limitations of sentinel site coverage and variability in data collection. Strengthening surveillance systems and expanding data collection to include asymptomatic cases and other key variables would provide a more comprehensive understanding of influenza dynamics in Cambodia. These findings serve to inform national and regional strategies for influenza prevention, control and preparedness.
